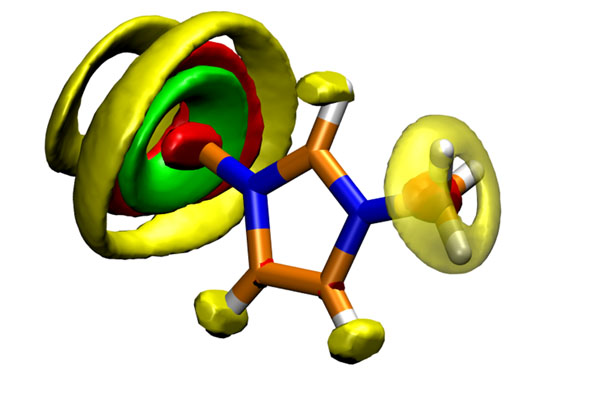# TRAVIS - a free analyzer and visualizer for Monte Carlo and molecular dynamics trajectories

**DOI:** 10.1186/1758-2946-4-S1-F1

**Published:** 2012-05-01

**Authors:** Martin Brehm, Barbara Kirchner

**Affiliations:** 1 Wilhelm-Ostwald-Institut für Physikalische und Theoretische Chemie, Universität Leipzig, Leipzig, Germany

## 

We present TRAVIS ("TRajectory Analyzer and VISualizer"), a free program package for analyzing and visualizing Monte Carlo and molecular dynamics trajectories [1]. The aim of TRAVIS is to collect as many analyses as possible in one program, creating a powerful tool and making it unnecessary to use many different programs for evaluating simulations. This should greatly rationalize and simplify the workflow of analyzing trajectories. TRAVIS is written in C++, open-source freeware and licensed under the terms of the GNU General Public License (GPLv3). It is easy to install (platform independent, no external libraries) and easy to use. On this poster, we present some of the algorithms that are implemented in TRAVIS - many of them widely known for a long time, but some of them also to appear in literature for the first time. All shown analyses only require a standard MD trajectory as input data.

**Figure 1:**